# Genome Editing in Model Strain *Myxococcus xanthus* DK1622 by a Site-Specific Cre/loxP Recombination System

**DOI:** 10.3390/biom8040137

**Published:** 2018-11-06

**Authors:** Ying-Jie Yang, Raghvendra Pratap Singh, Xin Lan, Cheng-Sheng Zhang, Yue-Zhong Li, Yi-Qiang Li, Duo-Hong Sheng

**Affiliations:** 1 State Key Laboratory of Microbial Technology, Microbiology Technology Institute, Shandong University, Qingdao 266237, China; yjyang@sdu.edu.cn (Y.-J.Y.); singh.dr.raghvendra@gmail.com (R.P.S.); lilab@sdu.edu.cn (Y.-Z.L.); 2 Marine Agriculture Research Center, Tobacco Research Institute of Chinese Academy of Agricultural Sciences, Qingdao 266101, China; zhchengsheng@126.com; 3 Research and Development Department, Biotechnology, Uttaranchal University, Dehradun 248007, India; 4 Department of Bio-Chemistry, Qingdao Technical College, Qingdao 266555, China; lanx@qtc.edu.cn

**Keywords:** Cre/loxP, large fragment deletion, *Myxococcus xanthus*, loxP spacer mutant, copper inducible promoter, *Escherichia coli*-*Myxococcus* shuttle plasmid, genome editing

## Abstract

*Myxococcus xanthus* DK1622 is a rich source of novel secondary metabolites, and it is often used as an expression host of exogenous biosynthetic gene clusters. However, the frequency of obtaining large genome-deletion variants by using traditional strategies is low, and progenies generated by homologous recombination contain irregular deletions. The present study aims to develop an efficient genome-engineering system for this bacterium based on the Cre/loxP system. We first verified the functionality of the native *cre* system that was integrated into the chromosome with an inducible promoter P*_cuoA_*. Then we assayed the deletion frequency of 8-bp-spacer-sequence mutants in loxP by Cre recombinase which was expressed by suicide vector pBJ113 or self-replicative vector pZJY41. It was found that higher guanine content in a spacer sequence had higher deletion frequency, and the self-replicative vector was more suitable for the Cre/loxP system, probably due to the leaky expression of inducible promoter P*_cuoA_*. We also inspected the effects of different antibiotics and the native or synthetic *cre* gene. Polymerase chain reaction (PCR) and sequencing of new genome joints confirmed that the Cre/loxP system was able to delete a 466 kb fragment in *M. xanthus*. This Cre/loxP-mediated recombination could serve as an alternative genetic manipulation method.

## 1. Introduction

The gliding Gram-negative myxobacteria, belonging to the delta division of Proteobacteria, are well known for social motility, multicellular differentiation, and large genomes of more than 9 Mb [1,2,3,4]. Myxobacteria can produce numerous secondary metabolites [5]. In addition to readily established microbial secondary metabolite producers like actinomycetes and fungi, research on myxobacteria as novel sources for natural products has gained attention. Within the genus *Myxococcus*, the species *Myxococcus xanthus* is one of the best-studied organisms among myxobacteria, since its lifecycle can be investigated under laboratory conditions. The potential of *M. xanthus* strain DK1622 [6] as a multiproducer of secondary metabolites has been investigated [7,8]. The *M. xanthus* genome-sequencing project [2] and subsequent in silico analysis revealed the presence of 24 putative biosynthetic gene clusters. Overall, 8.6% of the *M. xanthus* genome is dedicated to secondary metabolism, a higher percentage than that in various other established secondary metabolite producers. To date, six compounds have already been identified in DK1622 [9,10,11]. Remarkably, based on the data analyzed by transcriptomic and proteomic studies, the remaining unassigned pathways are active under standard conditions for the cultivation of DK1622 [12,13]. Furthermore, *M. xanthus* has been used as a heterologous expression host to express a secondary metabolite gene cluster from other difficult-to-handle myxobacteria or marine myxobacteria for producing macrolides such as epothilone, haliangicin, and disorazol [14,15,16,17].

Traditional strategies for obtaining gene-deletion variants in *M. xanthus* are mainly vector-based double-crossover methods, including incorporation of a kanamycin resistance gene and a counter-selectable genetic marker [18]. However, this large deletion event has extremely low frequency, and almost all of the progeny that were generated by this homologous recombination strategy contained irregular deletions because of the existence of a number of duplicated genes in huge genomes. The development of a technology to engineer recombination reactions using site-specific recombinases can significantly augment the tools available for molecular studies in *M. xanthus*.

Cre-loxP recombination is an important tool in molecular genetics. Cre recombinase from bacteriophage P1 recognizes a specifically autologous 34 bp loxP sequence, composed of an 8 bp spacer region flanked by two identical 13 bp inverted repeats [19]. When two loxP sites are in the same orientation on a linear DNA molecule, Cre-mediated intramolecular recombination results in the excision of the loxP-flanking region [20]. It does not require any host cofactor or accessory protein [21].

Here, we report the functional expression of native and codon-optimized *cre* genes cloned into different types of plasmids in *M. xanthus* DK1622, and we investigated the in vivo Cre-mediated recombination efficiencies. We found that P*_cuoA_*, an inducible promoter, has strong basal expression of the *cre* gene so that it cannot be used to integrate into chromosomes. Instead, *cre* expression by shuttle plasmid pZJY41 was confirmed to be a suitable method of manipulating the genome of *M. xanthus*. We constructed six plasmids to express the native *cre* gene or synthetic *cre* gene and applied these plasmids to delete a nearly 500 kb large fragment.

## 2. Results

### 2.1. Efficient Deletion of Lox-Flanking Gene Fragment by Native Cre Recombinase in M. xanthus

**Figure 1:**
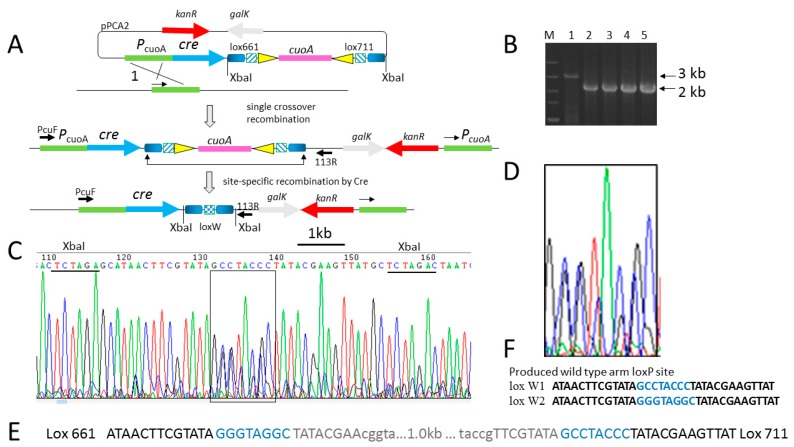
Native Cre recombinase can delete 1 kb fragment near the *cre* gene in *Myxococcus xanthus* DK1622. (**A**) Schematic diagram to verify native *cre* gene function by integration of the suicide plasmid pPCA2, which is simultaneously harboring the native *cre* gene and a 1 kb XbaI fragment flanked by two lox sites. Firstly, suicide plasmid pPCA2 was constructed to express the native *cre* gene under the copper-inducible promoter from DK1622. Following the *cre* expression cassette, the 1 kb XbaI fragment was cloned into the plasmid pPCA2 and flanked by two lox sites. A single crossover of homologous recombination at a 0.96 kb copper promoter occurred, and the strain was selected by kanamycin resistance. Secondly, a site-specific recombination occurred and deleted the 1 kb fragment *cuoA* covering the two lox sites. The promoter region as homologous arms as well is shown in light-green rectangles, respectively. The blue, gray, and red arrows represent the native *cre* gene, *galK* gene, and *kanR* gene, respectively. The short gradient blue rectangle, small squares with an oblique line, and small yellow triangle, represent the 13 bp wild-type arm, 8 bp crossover region, and 13 bp mutant arm of the lox site. (**B**) Identification of mutant strains by agarose gel electrophoresis of the polymerase chain reaction (PCR) product. PCR primers PcuF and 113R yielded the 2.0 kb positive band (indicated by the blue arrow) in four mutants (lanes 3–6), but yielded a 3.0 kb fragment in the negative control of DK1622 (lane 2). Lane 1 is a marker band. (**C**) Chromatograms of the 2.0 kb PCR product DNA sequence by primer 113R. A XbaI enzyme recognition site (TCTAGA) appeared at both ends of the new lox site, which contained two 13 bp wild-type arms. (**D**) Chromatograms of the expanded 8 bp region. Site-specific recombination occurred at the 8 bp crossover of lox661 and lox711, producing the mixed peak of lox sites GGGTAGGC and GCCTACCC. (**E**) The sequence of the 1 kb fragment covering two lox sites before being cut by native Cre recombinase is shown. The middle sequence of 1 kb was omitted. (**F**) The sequence of the new 13 bp wild-type arm of two lox sites.

To confirm whether the native Cre recombinase can delete the gene fragment in *M. xanthus* DK1622, we cloned the native *cre* gene from the 705 *cre* plasmid under the control of inducible promoter P*_cuoA_* to generate pBJ113-P*_cuoA_*-*cre* [22]. Two-mutant-loxP-flanking fragments of gene *cuoA* were ligated into the plasmid pBJ113-P*_cuoA_*-*cre* to generate plasmid pBJ113-P*_cuoA_*-cre-lox661-cuoA-lox711(pPCA2 in Figure 1A), two mutant arms and guanine- or cysteine-rich spacer sequences were used [23]. The plasmids were transformed into *Escherichia coli* top10 and verified by sequencing. The plasmids were introduced into DK1622 by means of electroporation to generate DK1622: pBJ113-P*_cuoA_*-cre-lox661-cuoA-lox711 and the correct integration was verified by PCR (Figure 1A). After preluding the Casitone-Tris (CTT) liquid medium with copper sulfate, primer PcuoF located at the end of promoter and primer pBJ113R designed outside of the multicloning site region of pBJ113 were used to amplify the total gDNA of the strain. As expected, 2.0 kb PCR products amplified, since 1 kb *cuoA* gene fragment flanking two loxP sites was deleted, and the 3 kb PCR product containing *cuoA* fragment was also amplified before induction of copper ion at the same PCR conditions (Figure 1B). After sequencing 2.0 kb PCR products of total DNA of mixed induced DK1622 pBJ113-P*_cuoA_*-cre-lox661-cuoA-lox711 cells, the results revealed that the 1.0 kb *cuoA* fragment was deleted, and two new loxP sites were produced, which suggested that the right or left spacer region was used alternatively in the ligation of two fragments by Cre recombinase (Figure 1C–F). Two molecules, loxPC(W1) or loxPG(W2), were produced and verified by the sequence chromatograph (shown in Figure 1C,D). Simultaneously, the mutant RE arm in the left lox site and the mutant LE arm in the right lox site were deleted; thus, two wild arms of the lox site appeared, indicating that native *cre* gene can be expressed and functioned in *M. xanthus* and can also recognize the high guanine- or cysteine-containing spacer sequences (Figure 1E). From the peak area of the sequence chromatograph, we roughly calculated the ratio of two molecules, that is, about 6:4 of loxPC:loxPG (Figure 1C,D). The ratio of these two spacers was calculated by the mixture of induced *M. xanthus* cells, which was isolated on CTT soft agar. After sequencing 10 single *M. xanthus* strains, we speculated that the ratio of the two molecules was 8:2 of loxPC:loxPG (Figure 1F). Because of the sequencing primer being located on the left arm of the vector, and these two mutant loxP sites being reverse-complement, the spacer GGGTAGGC was proved to be better than of the spacer GCCTACCC in the cutting and ligation reaction by Cre recombinase. It was also confirmed that Cre recombinase could recognize the spacer sequence by non-self-recombination.

### 2.2. Cre Expression Driven by the Inducible Promoter P_cuoA_ Was Found to Be Leaky during the Comparison of the Recombination Efficiencies of Different Lox Spacer Pairs by pBJ113-Cre Integration

**Figure 2:**
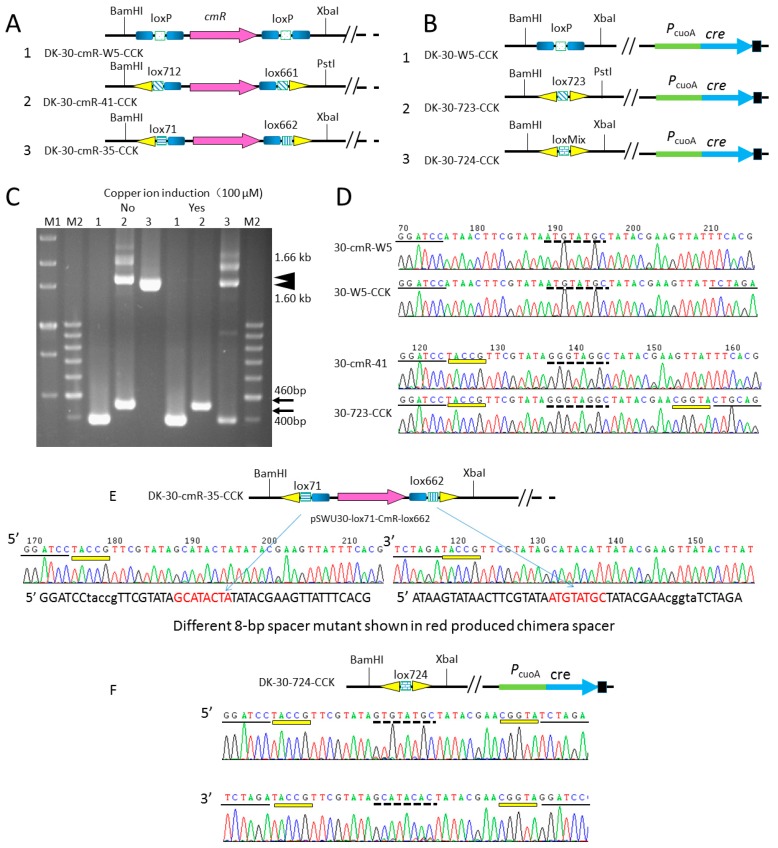
Identification of *cmR* deletion by *cre* gene integrated into chromosome and driven by copper inducible promoter with or without copper ion added using PCR and sequencing methods. (**A**) Diagrammatic sketch describing the *cmR*-flanking region of pSWU30 in strains DK-30-cmR-XX-CCK. *CmR* is shown by a purple arrow. Short gradient blue rectangle; small squares with different patterns filled and small yellow triangle represent 13bp wild type arm, different 8 bp crossover region and 13 bp mutant arm of lox site. There are enzyme recognition sites for BamHI, PstI or XbaI located at the outside of lox sites. (**B**) Diagrammatic sketch describing the *cmR*-deletion region of pSWU30 and the P*_cuoA_*-*cre* region in strains DK-30-XX-CCK. After *cmR* deletion by Cre recombinase, two identical wild type loxP would produce one wild type loxP. Two different lox sites lox712 and lox661 with one mutant arms, one wild type arm and same 8 bp crossover regions would produce one lox site lox723 with two mutant arms and the same 8 bp crossover region. Two different lox sites lox71 and lox662 with one mutant arms, one wild type arm and different 8 bp crossover regions would produce one lox site lox724 with two mutant arms and chimeric 8 bp crossover region. Light green arrow, light blue arrow and dark square represent the promoter P*_cuoA_*, the native *cre* gene and *rrnT1* terminator, respectively. (**C**) Identification of mutant strains by PCR without or with copper induction. After two rounds of electro-transformation into DK1622 by kanamycin and tetracycline selection, the single clones were cultured in Casitone-Tris (CTT) or CTT + CuSO_4_ (100 μM) liquid medium. Then, DNA was extracted and inspected by PCR. The PCR primers 113F and 113R were designed at the outside of multi-cloning site region of vector pSWU30. Since the longest undeleted fragment was 1.66 kb, the extension time of PCR was set to amplify 1.7 kb. LanesM1 and M2are marker. (**D**) Chromatograms of the PCR product DNA sequence by primer 113F. Enzyme recognition site BamHI, PstI or XbaI appeared at both ends of new lox site when *cmR* was absent. The 5-bp sequence at the mutant arms are marked in yellow thin rectangles. The sequences underlined are enzyme recognition sites. The 8-bp crossover region is also underlined with dotted line. (**E**) Diagrammatic sketch and chromatograms of *cmR*-containing region integrated into the genome with different 8bp crossover region shown in red.(**F**) Diagrammatic sketch and chromatograms of chimera crossover sequence. After the induction of *cre* gene, chimera crossover sequence was produced.

**Figure 3:**
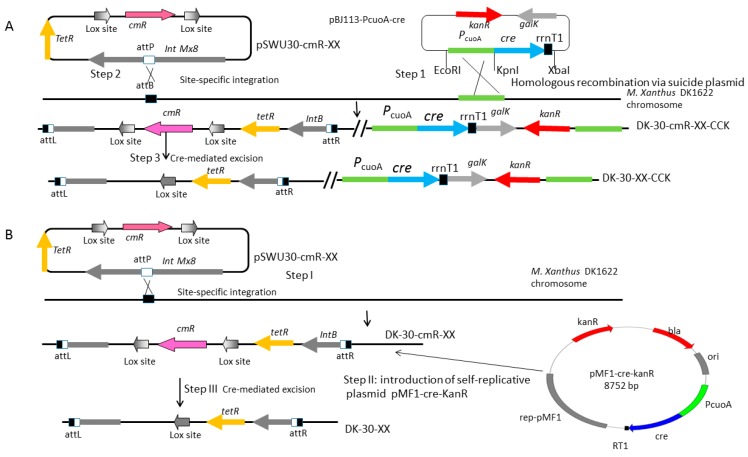
Diagrammatic representation of genome editing using the Cre/lox method. (**A**) The first strategy is to use suicide plasmid pBJ113 as a vector to integrate *cre* gene into the genome of *M. xanthus* DK1622 under the control of the copper inducible promoter. Step1, the suicide plasmid pBJ113-P*_cuoA_*-*cre* was first integrated into the genome by a single crossover of homologous recombination at the promoter region (shown in light green arrow) to obtain the strain DK-*cre* by kanamycin resistance (shown in red arrow). Step 2, pSWU30-cmR-XX series plasmids were integrated into the genome of strain DK-*cre* to obtain the strains DK-30-cmR-XX-CCK, screened by tetracycline resistance, respectively. Since pBJ113-P*_cuoA_*-*cre* and pSWU30-cmR-XX series plasmids carry the same *ori* region needed for the replication in *Escherichia coli* and the integration efficiency of pSWU30-cmR-XX series plasmids is higher than that of pBJ113-P*_cuoA_*-*cre*, we decided to integrate the series plasmid in step 2.The pSWU30-cmR-XX series plasmids contained a chloramphenicol resistance gene (*cmR*) and a constitutive promoter aphII, which are flanked by two lox sites in the multi-cloning site. Small gradient gray arrows represent the lox sites. *TetR* and *cmR* are shown in light orange arrow and purple arrow, respectively. Step 3, the expressed Cre recombinases bind to the lox sites, and the 8 bp crossover regions were recognized. Then, the double DNA strand was exchanged to produce two molecules when the orientation of two lox sites in the original one molecule chromosome are identical. In this case, the *cmR* gene was deleted. (**B**)The second strategy is to use self-replicative plasmid pZJY41 as a vector to express *cre* gene under the control of the copper inducible promoter. Step I, different from the first strategy, pSWU30-cmR-XX series plasmids were integrated into the genome of strain DK1622 to obtain the strains DK-30-cmR-XX with tetracycline resistance, respectively. This is due to the instability of pZJY41 vector in *M. xanthus*. Step II, pMF1-cre-kanR was transformed into DK-30-cmR-XX. The *kanR*+clones were screened at CTT+kan agar plates (40 μg/mL kanamycin). Step III, Cre recombinase bound the lox sites to delete the *cmR* gene by recombination. The strains were shaking in CTT+kan liquid medium with 20 μg/mL kanamycin (half of the normal concentration needed by pBJ113 integration) and without copper added for 48 h. Then the single clones were screened on CTT agar plate without antibiotics. Simultaneously, plasmid pMF1-cre-kanR was lost. Finally, series strains DK-30-XX were designated.

We constructed the *cre* expression system using the suicide plasmid pBJ113 under the P*_cuoA_* inducible promoter. A large number of mutations were investigated in the spacer region and it was observed that increased guanine content at all spacer positions except position 8 resulted in increased recombination [23]. To investigate the potential effects of the lox sites containing the mutant 8-bp spacer region in vivo, we constructed three plasmids containing different lox pairs (wild loxP/wild loxP, lox712/Lox661, and lox71/lox662), flanked a *cmR* gene to yield pSWU30-loxP-cmR-loxP, pSWU30-lox712-cmR-lox661, and pSWU30-lox71-cmR-lox662, respectively (Figure 2A, Table 1). The scheme for the construction is shown in Figure 3A. The three plasmids were electro-transformed into *M. xanthus* DK1622 to generate strains DK-30-cmR-W5, DK-30-cmR-41, DK-30-cmR-35, respectively. At last, we integrated pBJ113-P*_cuoA_*-*cre* in the above strains. We investigated the deletion efficiency of the *cmR* gene before and after the induction of copper sulfate by PCR amplification.

It was shown that P*_cuoA_* inducible promoter has strong basal expression based on the PCR results (Figure 2C). When copper ion was absent, one 0.40 kb band was observed in DK-30-cmR-W5-CCK and two 0.46 kb,1.6 kb fragments in DK-30-cmR-41-CCK by using primersV30-107F and V30-391R flanking the multi-cloning site of vector pSWU30, indicating the leaky expression driven by P*_cuoA_*, which cut the 1.1 kb *cmR* gene fragment. In the total DNA of DK-30-cmR-35-CCK, only one 1.6 kb fragment appeared which indicated that even if there is slightly leaky Cre recombinase, it was not enough to recognize the lox71-cmR-lox662 pairs with two different normal 8 bp spacers. When 100 μM copper ion were added, cm resistance gene (*cmR*) could be completely deleted in DK-30-cmR-W5-CCK and DK-30-cmR-41-CCK to produce strain DK-30-W5-CCK and DK-30-41-CCK (Figure 2B,D). Approximately equal quantity of PCR products appeared in the strain DK-30-cmR-35-CCK which contained two different normal GC content spacers (Figure 2B,E,F). Irrespective of the presence or absence of 100 μM copper sulfate, *cmR* genes flanked by wild type loxP, were deleted. *cmR* genes flanked by lox712 and lox661 were partially deleted when it was not induced (Figure 2C). However, *cmR* genes flanked by lox71 and lox662 were partially deleted even if copper ion was added into CTT liquid medium. The fragments containing the produced lox site were verified by sequencing, indicated that copper inducible promoter has leaky expression to edit lox site on genome in the cell of DK1622 and suggesting the Cre recombinase have different cleavage efficiency at different lox pairs.

### 2.3. Cre Expression from pZJY41-Self-Replicative Plasmid Has HigherEfficient Recombination of Different Lox Pairs

**Figure 4:**
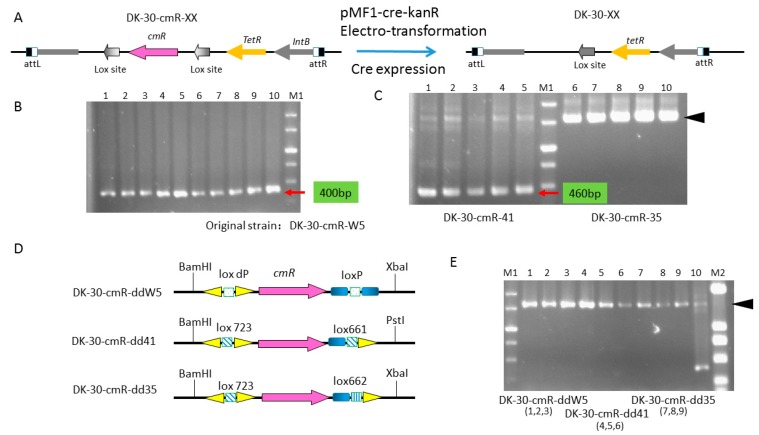
Identification of *cmR* deleted by pMF1-cre-kanR without copper sulfate addition using PCR and sequencing methods. (**A**) Diagrammatic sketch describing the process of strains DK-30-cmR-XX to DK-30-XXby pMF1-cre-kanR.Light orange and purple arrows represent *tetR* gene and *cmR* gene, respectively. The lox sites before deleting the *cmR* gene are shown in gradient gray. The lox site produced is shown in dark gray after the electro-transformation of pMF1-cre-kanR and the expression of *cre* gene. (**B**) Identification of mutant strains by agarose gel electrophoresis of PCR product of original strain DK-30-cmR-W5after the electro-transformation of pMF1-cre-kanR. Lane1–10 show the band of the deleted region of pSWU30 vector sequence containing the produced loxP by primer V30-41F and V30-391R. (**C**) Identification of mutant strains by agarose gel electrophoresis of PCR product of original strain DK-30-cmR-41 and DK-30-cmR-35 after the electro-transformation of pMF1-cre-kanR. Lanes 1–5 show the band of the deleted region of pSWU30 vector sequence containing the produced lox723 by primer pair V30-41F and V30-391R in the original strain DK-30-cmR-41. Lanes 6–10 show the band of the undeleted *cmR* region of pSWU30 by primer pair V30-41F and V30-391R in the strain DK-30-cmR-35. (**D**) Diagrammatic sketch describing the cmR-flanking region of pSWU30 in strains DK-30-cmR-ddXX. *CmR* is shown in purple arrow. Short gradient blue rectangle, small squares with different patterns filled and small yellow triangle represent 13-bp wild type arm, different 8-bp crossover region and 13 bp mutant arm of lox site. There are enzyme recognition sites for BamHI, PstI or XbaI located at the outside of lox sites. (**E**) Agarose gel electrophoresis of the PCR product by primer pair V30-41F and V30-391R to strain DK-30-cmR-ddW5 (lanes 1–3), DK-30-cmR-dd41(lanes 4–6) and DK-30-cmR-dd35 (lanes 7–9) after the electro-transformation of pMF1-cre-KanR.Lane 10 is the control of DK-30-cmR-41. M1 and M2 are the markers.

Because of the leaky expression of Cre recombinase driven by the inducible promoter, we used *E.coli-Myxococcus* shuttle plasmid pZJY41 to develop a Cre/lox recombination system in DK1622 (Figure 3B and Figure 4A). To verify the functionality of Cre recombinase cloned into pZJY41, we introduced the pMF1-cre-kanR into strain DK-30-cmR-W5, DK-30-cmR-41and DK-30-cmR-35, respectively (Figure 4A). After 6 days of cultivation on a CTT+kan agar plate, we transferred the kanamycin resistant clones onto new CTT+kan agar plates for two days and cultivated the clones in CTT liquid medium with 20 ug/ml of kanamycin. Then, the total DNA was extracted and amplified by primers V30-107F and V30-391R. Like Cre recombinase expressed by pBJ113 integration, we could not obtain the 1.6 kb fragment containing the *cmR* gene from strain DK-30-CmR-W5, suggesting that *cmR* genes flanked by wild type loxP were all deleted even if copper ion was not added (Figure 4B). For the strain DK-30-cmR-41, strong 0.46 kb band and slight 1.6 kb band appeared, indicating that cleavage efficiency by *cre* expression cloned in pZJY41 was better than that of pBJ113(Figure 4C). For DK-30-cmR-35, similar to pBJ113, in the absence of copper ion, only 1.6 kb band appeared, suggesting that the basal expression could not recognize the mutant lox which contained two different normal GC spacers (Figure 4C, right). Based on the PCR results, we further confirmed that the inducible promoter P*_cuoA_* has strong basal expression and found the *cre* expression in pZJY41 has higher cleavage efficiency than that in pBJ113.

To confirm the double mutant arms of lox723, which were produced by the lox712 and lox661 pair, not to affect the next step of recombination, we constructed mutant DK-30-cmR-ddW5, DK-30-cmR-dd41, DK-30-cmR-dd35 (Figure 4D). Based on the PCR results, no recombination was found between the two mutant arms of lox723 and the other loxPs, even using the most favored space sequence GGGTAGGC (Figure 4E) and adding 100 μM copper into CTT+kan (10 μg/mL) liquid medium.

### 2.4. Comparison of the Recombination Efficiency in Different Expression Type (pBJ113 and pZJY41) with Different Lox Pairs

**Figure 5:**
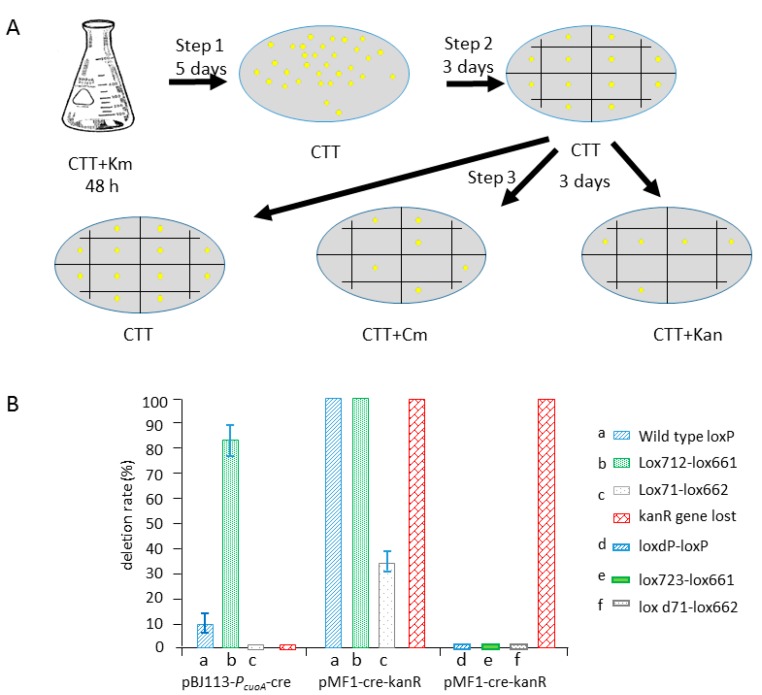
Deletion efficiency of *cmR* gene flanked with different lox pairs by *cre* gene expressed on chromosome or plasmid via single clone isolation on CTT+antibiotics agar plate. (**A**) Workflow of screening cm-deleted clones by the integration of pBJ113-P*_cuoA_*-*cre* and pMF1-cre-kanR in *M. xanthus*. Step 1, DK-30-cmR-XX or ddXX-CCK and DK-30-cmR-XX∷pMF1-cre-kanR was cultured in CTT+kan liquid medium (40 μg/mL, 20 μg/mL) without copper sulfate addition for 48 h at 30 °C respectively. Then, the broth was diluted and spread onto a CTT agar plate and culture for 5 days at 30 °C. Step 2, When the single clones appeared, and then transferred onto new CTT agar plates for 3 days at 30 °C. Step 3, after 3 days of culture, transfer onto CTT, CTT+cm (chloromycetin), CTT+kan agar plates, respectively, to check the growth or not. (**B**) Deletion efficiency of *cmR* gene flanked with different lox pairs by *cre* gene expressed on the integrated chromosome or self-replicative plasmid via single clone isolation on CTT+cm agar plates. Line a–f represents the different lox pairs flanking the chloramphenicol resistance gene integrated into the genome of DK1622 by the pSWU30-cmR series plasmids: pSWU30-cmR-W5, pSWU30-cmR-41, pSWU30-cmR-35, pSWU30-cmR-ddW5, pSWU30-cmR-dd41 and pSWU30-cmR-dd35, respectively. *Cre* gene was integrated into genome of DK1622 by pBJ113-P*_cuoA_*-*cre* and expressed on the genome or expressed on self-replicative plasmid pMF1-cre-kanR. Fifty clones of every genotype were picked (three repetitions).

To accurately determine the ratio of deleted 0.46 kb and undeleted 1.6 kb in *M. xanthus* cells, we screened the two kinds of clones, one which deleted the loxP-flanking cm resistant gene in the site-specific vector pSWU30 and other, which did not. The workflow of the experiment has been illustrated in the Figure 5A. The pBJ113-*cre* expression loxP-containing strain DK-30-cmR-W5-CCK, DK-30-cmR-41-CCK and DK-30-cmR-35-CCK were cultivated in the CTT+kan liquid for 48h to the log phase. Since, pZJY41-*cre* containing strains grown slowly in CTT+kan liquid medium, we added half of the concentration of the kanamycin, (20 μg/mL). After that, we spread the culture onto CTT agar plates and cultivate it for 5 days. After appearance of single clones, we transferred these single clones onto new plate. Finally, these clones were transferred onto CTT agar plate, CTT+cm agar plate, CTT+kan agar plate, respectively (Figure 5A).

Comparison of the PCR results for the completeness of 0.46 kb PCR fragment under the induction by copper or not, only 9% clones were confirmed to delete the *cmR* gene and did not find any induction by copper in wild-type loxP (two native arms and two same native spaces). InDK-30-cmR-35 (two mutant arms and one native space and one different reverse mutant space) only 1% clones were confirmed to delete the *cmR* gene. In the strain DK-30-cmR-41 (two mutant arms LE, RE and two same mutant high GC space), 83% clones were confirmed to delete the *cmR* gene, indicating that high GC-content fragment can be efficiently recognized by Cre recombinase in vivo (Figure 5B).

Based on *cre* expression on pZJY41, the screening of single clones was performed by the same method illustrated before (Figure 5A). It was found that a wild type loxP/loxP and Lox712/Lox661 pair-containing strain DK-30-cmR-W5, DK-30-cmR-41 completely lost the *cmR* after cultivation for 48 h in CTT+kan liquid culture (20 μg/mL) by expression of *cre* cloned in pZJY41(Figure 5B). One third of the Lox71/Lox662 pair-containing strain DK-30-cmR-35 lost *cmR* (Figure 5B). The results indicated that *cre* expression in pZJY41 had higher efficiency than that in the chromosome. Most importantly, after twice restreaking this on a CTT agar plate (without kanamycin), plasmid pMF1-cre-kanR disappeared; thus, it is convenient to integrate loxPat next generation. Not only because of the higher activity of *cre* recombinase but also the easily lost characteristic of pMF1-cre-kanR, it is the best choice to develop the cre/lox system in *M. xanthus*.

In order to confirm that the double mutant arms of loxP/Lox723 do not affect the next step of recombination, mutant DK-30-cmR-ddW5, DK-30-cmR-dd41 and DK-30-cmR-dd35 were also constructed, which could be introduced by pMF1-cre-kanR.Based on the PCR results and isolation of *cmR* clones, we did not find the recombination by double mutant arms of lox723 with other loxP even if the high efficiency space sequence GGGTAGGC was used (Figure 5B).

### 2.5. Construction of a Series of Self-Replicative Plasmids for the Expression of Native Cre Gene or Artificial Cre Gene in M. xanthus by Different Antibiotics Markers

Using the synthetic *cre* (artificially synthesized) gene with a GC content of 67.7% optimized for expression in high-GC bacteria, Fedoryshyn and their colleagues have removed an apramycin resistance gene flanked by loxP sites from the chromosome of *Streptomyces slividans* with 100% efficiency. However, the loss frequency of the apramycin resistance gene was less than 1% by the native *cre* gene [25]. So we synthesized the *cre* gene with a GC content of 67.1% optimized for expression in *M. xanthus*.

Furthermore, to facilitate the choice of the *cre* expression vector, we constructed a series of plasmids by using two other antibiotic resistance genes (Figure S1). All the plasmids contained a native *cre* gene or artificial *cre* gene driven by the P*_cuoA_* promoter and terminated byrrnT1 terminator from *E. coli*. Three of these vectors, pMF1-cre-kanR, pMF1-cre-tetR, pMF1-cre-cmR, harboring *kanR*, *tetR*, *cmR* marker, respectively, were constructed by using the native *cre* gene based on the self-replication plasmid pZJY41. The other three, pMF1-cre(a)-kanR, pMF1-cre(a)-tetR, pMF1-cre(a)-cmR were constructed by using an artificial synthetic *cre* gene from the above three plasmids. Since a low concentration of chloromycetin (cm) could cause the genetic instability (unpublished data), confirmation of the existence of the *cre* gene must be conducted by PCR when pMF1-cre-cmR or pMF1-cre(a)-cmR were used.

### 2.6. Deletion of 468 kb Large Fragment Covering Yellow Pigment Gene Cluster and Siderophore Gene Clusters

**Figure 6:**
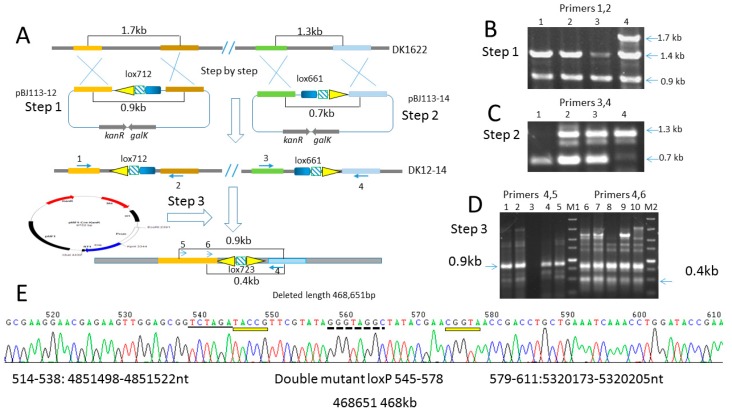
Deletion of large fragments in *M. xanthus* DK1622. (**A**) Schematic diagram to delete the large fragment in DK1622. Firstly, pBJ113-12, which contains left lox site lox712, was integrated into the genome of DK1622 by kanamycin selection; the second crossover occurred by galactose-added culture to obtain strain DK 12 (confirmed in Figure 6B). Secondly, pBJ113-14, which contains right lox site lox661, was electro-transformed into the genome of DK 12 to obtain strain DK 12-14by the same method (confirmed in Figure 6C). Thirdly, *cre*-expressing self-replicative plasmid was electro-transformed into strain DK 12-14 and large fragment was deleted and confirmed in Figure6D. (**B**) Identification of double crossover strains DK 12 by agarose gel electrophoresis of the PCR product of original strain DK1622 after the electro-transformation of pBJ113-12. Lanes 1–3 show 0.9 kb band of the double crossover strains DK 12 by primer 1 (S12-1237F) and2 (S12-2117R). Lane 4 shows the mixture from the wild type (1.7 kb) and mutant type (0.9 kb). 1.4 kb band is amplified as non-specific product. (**C**) Identification of double crossover strains DK 12-14 by agarose gel electrophoresis of PCR product of original strain DK 12 after the electro-transformation of pBJ113-14. Lane1 shows 0.7 kb band of the double crossover strains DK 12-14 by primer 3 (S14e-837F) and 4 (S14e-2188R). Lanes 2–3 show the mixture of the original type DK 12 (1.3 kb) and mutant type (0.7 kb). Lane 4 is the band from original type DK 12 (1.3 kb). (**D**) Identification of the deletion of large fragmented by agarose gel electrophoresis of PCR product after the electro-transformation of cre-expressing self-replicative plasmid into DK12-14. Lanes 1–5 (except lane 3) show 0.9 kb band amplified by primer 5 (S12-LPF1) and 4 (S14e-2188R). Lanes 6–10 show the PCR products amplified by primers 6 (S12-LPF2) and 4 (S14e-2188R), which contained many non-specific products. The 0.4 kb specific product appeared but was slight. M1 and M2 are the band markers. (**E**) Chromatograms of the 0.9 kb PCR product DNA sequence by primer 5 (S12-LPF1). In the sequencing data, the sequence 514–538 (25 bp) is identical to that 4,851,498–4,851,522 in DK1622. Sequence 539–544 is the enzyme recognition site XbaI shown in underline and incorporated in the primer sequence. Following that, there is a double mutant arm lox site lox723 (545–578) produced. The sequence 579–611 is identical to that 5,320,173–5,320,205 in *M. xanthus* DK1622. Sequence 5’TACCG, which is the signature sequence of mutant arm, appeared at the both end of new lox site lox 723 when the large fragment was deleted. The 5 bp sequence at the mutant arms are marked in yellow thin rectangles. The 8 bp crossover region also underlined with dotted line. Based on the result of BLAST to the genome of DK1622, a 468,651 bp fragment was deleted.

To confirm the function of constructed six *cre*-expression plasmids in *M. xanthus* DK1622, we deleted a large fragment covering yellow pigment Myxococcus xanthene biosynthetic gene cluster and siderophore biosynthetic gene clusters from MXAN_3982 to MXAN_4322, (about 468 kb). We first inserted the two highly efficient mutant loxP pair, lox712 and lox661 into chromosome position4851522 and 5320173 by two homologous arm suicide plasmids pBJ113-12 and pBJ113-14 via two crossover recombinations, respectively (Figure 6A–C).

Using the pMF1-cre-kanR *cre* expression system, we can get the PCR fragments by both sides of the primer pairs of the produced loxP site, primers 5,4:S12-LPF1, S14e-2188R (0.9 kb); primers 6,4:S12-LPF2, S14e-2188R (0.4 kb); we obtained the 0.9 kb fragment from primers 4 and 5 and 0.4 kb fragment from primers 4 and 6 (Figure 6D). To further confirm the deletion of large fragment and the product of double mutant lox site, we sequenced the two fragments and speculated that the mutant sequence of lox723 (taccgTTCGTATAGGGTAGGCTATACGAAcggta) (Figure 6E) was produced which have indicated that the Cre recombinase gene in plasmid pMF1-cre-kanRwas expressed and deleted in the large fragment in *M. xanthus*. We have also used the codon-optimized *cre* gene being cloned into plasmids pMF1-cre(a)-kanR, pMF1-cre(a)-tetR and pMF1-cre(a)-cmR. As a result, we confirmed that the constructed plasmid can express Cre recombinase and the large fragment was deleted.

## 3. Discussion

In this study, we developed a method to delete large fragments based on the Cre/loxP system and constructed six *E.coli-M. xanthus* shuttle plasmids for *cre* expression in *M. xanthus* DK1622. In the Cre/loxP recombination system, the control of the expression of the*cre* gene is a key challenge in the development of the method. There are two choices to control the expression of the *cre* gene. The first choice is to use the inducible promoter to control the expression. The second choice is to use the self-replicative plasmid, which can be readily introduced or lost to prevent the expression of the *cre* gene when not needed. Since the strain containing pZJY41 grows slowly [26], we firstly attempted to select an inducible promoter in *M. xanthus* DK1622. Unfortunately, the recently discovered copper inducible promoter was not suitable for controlling the expression of the *cre* gene. Although we have successfully controlled the expression of cas9 by a copper inducible promoter in *M. xanthus* DK1622 [27], the slight basal expression of *cre* could recognize and digest the loxP-containing fragments. However, it is not clear yet whether the basal expression driven by P*_cuoA_* was caused by the contaminants of chemicals used for the media or not. We found the social motility is slightly restored in the reference Figure 6B when SA4100 cells are spotted onto plates with 0 μM copper concentration, compared to DK10416 [22]. Therefore, we used our constructed pZJY41 and constructed six *E.coli-M. xanthus* shuttle plasmids for native or artificial *cre* expression in *M. xanthus* DK1622 by using *kanR*, *tetR* and *cmR* resistance genes.

Besides the *cre* expression pattern, the sequence of loxP sites is another crucial factor to influence the deletion efficiency. Based on the in vitro results, 8-bp spacer mutant type of lox site pair could affect the deletion efficiency [23]. To compare recognition to 8-bp spacer mutant type of lox site pair in vivo, we designed four lox sites pairs flanking *cmR* resistance gene to investigate the deletion efficiency by *cre* gene integrated into chromosome by pBJ113 homologous recombination and by self-replicative plasmid pZJY41. No matter which kinds of *cre* expression pattern, lox pairs which contain high guanine content 8 bp spacer pair have the highest recognition by Cre recombinase; this is consistent with the results in vitro published by Missirlis et al*.* [23] and we firstly proved it in vivo. The pairs of loxP sites (for example, lox71 and lox66) which contained 13 bp wild type arm and 13 bp mutant arm was also proven to be suitable to produce double mutant 13 bp arms of lox sites which were proven not to be recognized again in *M. xanthus*.

Based on our results, the utilization of pZJY41 in *M. xanthus*, especially in large fragment deletion and construction of the chassis of bacteria cell was evaluated. Although the genetic operation by this Cre/loxP system was time-consuming, compared with our developed CRISPR/Cas9 system and well used galactose counter-selection, it has nearly 100% deletion efficiency. Extra chromosomal autonomously replicating genetic materials are normally absent from myxobacterial cells. Up to now, pMF1, originally discovered from *Myxococcus fulvus* 124B02 in 2008 [26], is the one and only endogenous plasmid that is able to replicate autonomously in myxobacterial cells. Autonomously replicating plasmid pMF1 provides a new tool for genetic manipulation in *Myxococcus*. However, there are some disadvantages of pZJY41. For example, it does not normally transcribe the kanamycin resistance gene cloned in the shuttle plasmid. We do not know why the strains carrying many copies of the kanamycin resistance gene plasmid pMF1 did not grow normally. Due to the uncertain expression level of some genes cloned into the free plasmids, it might hinder the utilization of plasmid-based expression.

## 4. Materials and Methods

### 4.1. Bacterial Strains, Growth Conditions, Plasmids, and Oligonucleotides

The *M. xanthus* and *E. coli* strains used in this study have listed in Table 2. *M. xanthus* strains were grown at 30 °C in liquid Casitone-Tris (CTT) medium with vigorous shaking. When necessary, media were supplemented with kanamycin (40 µg/mL), tetracycline (15 µg/mL), chloramphenicol (10 µg/mL), or copper sulfate (100 µM). *E. coli* strains were grown at 37 °C in Luria–Bertani medium, which was supplemented with ampicillin (100 µg/mL), kanamycin (20 µg/mL), tetracycline (10 µg/mL) or chloramphenicol (10 µg/mL) when needed. The plasmids used in this study are listed in Table 3. DNA manipulation and cloning was performed according to the standard protocols (Sambrook et al. 1989). Laboratory constructed plasmids were verified by PCR and DNA sequencing. All oligonucleotides have been mentioned in the Supplementary Table S1. pNG10A and pMAT3, which were kindly provided by José Muñoz-Dorado (University of Granada, Granada, Spain), were used as template DNA to amplify the *cuoA* promotor and *tetR* gene.

### 4.2. Construction of Lox-CmR-Lox-Containing Plasmids

To construct pSWU30-cmR-XX series plasmids, we amplified the 1.1 kb chloramphenicol resistance gene attached with the native promoter using the different type of lox site primer pairs from plasmid pKK232-8 [32]. Wild type loxP pair was introduced by the forward primer CmR-WloxPBamHI and reverse primer CmR-WloxPXbaI, respectively. Then, the PCR fragment was digested with BamHI+XbaI to ligate into pSWU30 [31] directly to give plasmid pSWU30-cmR-W5. Primer pair CmR-Lox71F BamHI and CmR-Lox662R XbaI was used to construct the plasmid pSWU30-cmR-35 as the method mentioned above. As to the construction of pSWU30-cmR-41, 8 bp high GC-content crossover region lox pair was introduced by the forward primer CmR-loxPFClaI and reverse primer CmR-loxPREcoRV; then the fragment was digested with ClaI+EcoRV and ligated into the SmaI linearized pZJY41 to give the plasmid pZJY41-cmR-lox. Finally, the backbone of pZJY41-cmR-lox was changed to give plasmid pSWU30-cmR-41 by enzyme BamHI+XbaI. Since the BamHI+XbaI fragment of *cmR* contains the multi-cloning site sequence of pZJY41, it can be 60 bp longer than those of pSWU30-cmR-W5 and pSWU30-cmR-35.

To construct series of plasmids containing 13 bp arm double mutants inside the one lox site, primer pair DDW5F2 and DDW5R was used to amplify pSWU30-cmR-W5. After phosphorylation and self-ligation, we obtained the plasmid pSWU30-cmR-ddW5. By the same method, we obtained the plasmid pSWU30-cmR-dd41and pSWU30-cmR-dd35 by primer pairs, DD41F and DD41R, DD35F2and DD35R, respectively.

### 4.3. Construction of the Series of Native Cre Expression Plasmids

The native *cre* gene was amplified from the template of the 705-*Cre* plasmid [33] with primer CreF2 and primer CreBT1R containing KpnI enzyme recognition site, and XbaI enzyme recognition site and rrnT1 terminator sequences, respectively. The *cre* gene fragment accompanied with the rrnT1terminator was digested by KpnI+XbaI and ligated to pUC19 [29] to give pUC19-cre. The 0.96 kb copper inducible promoter sequence was amplified from plasmid pNG10A with primer pair PcuF and PcuR, then digested by EcoRI+KpnI and ligated to pUC19-*cre* to obtain pUC19-P*_cuoA_*-cre. The backbone of pUC19-P*_cuoA_*-*cre* was changed into pBJ113 [30] by EcoRI+XbaI digestion and ligation to give plasmid pBJ113-P*_cuoA_*-*cre*. Since the 0.96 kb copper-inducible promoter sequence was originated from the genome of DK1622, the plasmid pBJ113-P*_cuoA_*-*cre* can be inserted into the genome of DK1622 via homologous recombination. To obtain the expression vector pMF1-cre-kanR, plasmid pZJY41 was digested with EcoRI+XbaI and ligated with the EcoRI+XbaI fragment of pUC19-P*_cuoA_*-cre.

For convenient use of the vector pMF1-cre-kanR in combination with other antibiotic-resistant vectors in *M. xanthus*, we replaced the kanamycin resistant gene (*kanR*) with the tetracycline resistance gene (*tetR*) or the chloramphenicol resistance gene to generate pMF1-cre-tetR and pMF1-cre-cmR. First, we amplified the backbone of pMF1-cre-kanR with primer pair V41-3979F and V41-4798R, located upstream of the *kan* gene and downstream of *bla* gene in pMF1-cre-kanR, respectively. The *tetR* gene was amplified with primer pair Tet1172F and Tet2644R using pMAT3 as template. The *cmR* gene was amplified with primer pair CmRF and CmRR using pKK232-8 as template. Both amplicon and resistant gene fragments were precipitated, digested by SmaI and ligated, then electroporation to *E. coli* Top10. The plasmids pMF1-cre-tetRand pMF1-cre-cmR were confirmed by restriction enzyme digestion and sequencing.

### 4.4. Construction of the Series of Artificial Synthetic Cre Expression Plasmids

Codon optimized artificial *cre* (abbreviate cre(a)) was designed by the method shown in the homepage www.kazusa.or.jp. On the basis of the principle of codon optimization, only less than 10% rare codons in *M. xanthus* were changed into high GC-content codon. The *cre(a)* gene attached with the rrnT1 terminator was synthesized (shown in additional files), NdeI site was placed at front of *cre*(a) gene, EcoRV and EcoRI placed at the end of terminator, and finally was cloned in the SmaI site of pUC57 vector to give the plasmid pUC57-*cre*(a).

The construction of synthetic *cre* expression plasmids was performed by red/ET recombination. Firstly, two primer pairs which contained 20 bp short homologous nucleotides were used to obtain two PCR fragment. Specifically, primer pair CuMcrF and McrCuR was used to amplify the 1.0 kb artificial *cre* gene used pUC57-M*cre* as a template. Primer pair Mcr41F and 41McrR was used to amplify the backbone of pMF1-cre-kanR, pMF1-cre-tetR and pMF1-cre-cmR to produce three PCR fragments which contained kanamycin resistance gene, tetracycline resistance gene and the chloramphenicol gene, respectively. Secondly, the *cre*(a) fragment and the three backbones of the pMF1-resistance-gene which contained the two *ori* regions and one resistant gene were mixed, then transformed into *E. coli* GB05dir in order to generate the homologous recombination of these two fragments. Finally, the plasmids pMF1-cre(a)-kanR, pMF1-cre(a)-tetR and pMF1-cre(a)-cmR were confirmed by restriction analysis and sequencing.

### 4.5. Construction of Homologous Arms Plasmids pBJ113-12 and pBJ113-14

To construct the suicide plasmid pBJ113-12 to insert lox712 into the upstream region of the siderophores synthesis gene cluster, we used conventional cloning method. Firstly, we amplified the two homologous arms by two primer pairs S12uu-130F and S12uu-1638R2, S12uF2 which contains lox712 and S12uR2. Then, these were doubly digested by EcoRI and XbaI, XbaI and HindIII, respectively and ligated into pBJ113 stepwise. Finally, the clones were confirmed by double digestion and sequencing.

For the construction of suicide plasmid pBJ113-14 to insert lox661 into the downstream region of DK xanthene biosynthetic gene cluster, we used Red/ET recombination method. Briefly, we amplified the two homologous arms by two primer pairs S14E-451F which contains the pBJ113 vector multi-cloning site sequence and S14E-1457R, S14E-2057F which contains lox661 and the overlapped sequence of S14E-1457R and S14E-3649R which contains the pBJ113 vector multi-cloning site sequence. After purification of these two PCR products and linearized pBJ113 vector, these three fragments were simultaneously transformed into arabinose-induced *E. coli* GB05dir[z]. Finally, the plasmids were confirmed by double digestion and sequencing.

### 4.6. Construction of the Mutant Strains: DK-30-cmR-XX-CCK Series Strains and DK-30-cmR-XX Series Strains

Site-specific integrative plasmid pSWU30 and suicide plasmid pBJ113 contain the same *ori* region sequence (about 1.0 kb). Simultaneously, the integration efficiency by pSWU30 is more than tenfold that of homologous arm-containing pBJ113. To avoid the occurrence of homologous recombination at the *ori* region when we needed to use these two plasmids to integrate the same genome of DK1622 step by step, we adopted a strategy as described: firstly, the pBJ113 vector was used by kanamycin selection, and then used the pSWU30 vector by tetracycline selection. Therefore, to construct the mutant strains DK-30-cmR-XX-CCK, we firstly integrate the pBJ113-P*_cuoA_*-*cre* into DK1622 to give the strain DK-P*_cuoA_*-cre. Then pSWU30-cmR-XX series plasmids were electro-transformed into strain DK-P*_cuoA_*-creto obtain the series strains DK-30-cmR-XX-CCK, respectively. This is the process of obtaining *cmR* deletion strains and verifying the deletion efficiency via different types of mutant lox pairs by *cre* gene integrated into the chromosome genome and expressed by copper inducible.

To investigate the deletion efficiency of different lox pairs by Cre recombinase expressed on shuttle plasmid pZJY41 in DK1622, we first construct the series strains DK-30-cmR-XX containing different lox pairs. A series of plasmids pSWU30-cmR-XX was electro-transformed into strain DK1622 to obtain the series strains DK-30-cmR-XX, respectively.

### 4.7. Construction of the Lox Site Containing Mutant Strain DK-12-14

To obtain the strain containing double mutant lox sites at different regions of the chromosome of DK1622, we used the suicide plasmid pBJ113-12which contained two homologous arms and lox712 designed between the two homologous arms, integrated into the genome of DK1622 by kanamycin selection. After confirming the correct integration by PCR amplification, then the double crossover occurred on CTT+galactose agar plates; the correct recombination clones were obtained by PCR amplification and sequencing. After purification, we got the lox712-containing strain DK-12 at the desired position. By the same method, we electro-transformed the plasmid pBJ113-14 into the strain DK-12 to obtain the strain DK-12-14 which contains a double mutant-type lox pair in the genome of DK1622 and at about the 468 kb region was flanked by the two lox sites.

## Figures and Tables

**Table 1:** loxP sequences used in this study.

Name of LoxP	Left Arm	Spacer	Right Arm	Reference
Wild loxP	ATAACTTCGTATA	GCATACAT	TATACGAAGTTAT	[19]
lox 66	ATAACTTCGTATA	GCATACAT	TATACGAAcggta	[24]
lox 71	taccgTTCGTATA	GCATACAT	TATACGAAGTTAT	[24]
lox 661	ATAACTTCGTATA	GGGTAGGC	TATACGAAcggta	This study
lox 711	taccgTTCGTATA	GCCTACCC	TATACGAAGTTAT	This study
lox PC(W1)	ATAACTTCGTATA	GCCTACCC	TATACGAAGTTAT	This study
lox PG(W2)	ATAACTTCGTATA	GGGTAGGC	TATACGAAGTTAT	This study
lox 712	taccgTTCGTATA	GGGTAGGC	TATACGAAGTTAT	This study
lox 662	ATAACTTCGTATA	TAGTATGC	TATACGAAcggta	This study
lox 723	taccgTTCGTATA	GGGTAGGC	TATACGAAcggta	This study
lox dp	taccgTTCGTATA	GCATACAT	TATACGAAcggta	This study

**Table 2:** Strains used in this study.

Strains	Genotype	Reference or Source
*E. coli*		
*E. coli* TOP10	F^–^*mcr*A Δ(*mrr*-*hsd*RMS-*mcr*BC) Φ80*lac*ZΔM15 Δ*lac*X74 *rec*A1 *ara*D139 Δ(*araleu*) 7697 *gal*U*gal*K*rps*L (StrR) *end*A1 *nup*G	Thermo Fisher Scientific (MA, USA)
*E. coli* DH5α	F^–^ Φ80*lac*ZΔM15 Δ(*lac*ZYA-*arg*F) U169 *rec*A1 *end*A1 *hsd*R17 (rK–, mK+) *pho*A*sup*E44 λ– *thi*-1 *gyr*A96 *rel*A1	Thermo Ffisher Scientific (MA, USA)
*E. coli* GB05	F-mcrA ∆(mrr-hsdRMS-mcrBC) φ80lacZ∆M15 ∆lacX74 recA1 endA1 araD139 ∆(ara, leu)7697 galUgalK λ rpsLnupGfhuA::IS2 recET redα, phage T1-resisten	[16]
*E. coli* GB05-dir	GB2005, araC-BAD-ETγA	[28]
*M. xanthus*		
DK1622	Wild type	[6]
DK-WGC	DK1622::pBJ113-P*_cuoA_*-*cre*-lox661-cuoA-lox711, Kan	this study
DK-WG	DK1622::pBJ113-P*_cuoA_*-*cre*-loxW1, Kan	this study
DK-WC	DK1622::pBJ113-P*_cuoA_*-*cre*-loxW2, Kan	this study
DK-30-cmR-W5	DK1622::pSWU30-loxP-cmR-loxP, TetR, CmR	this study
DK-30-cmR-41	DK1622::pSWU30-lox712-cmR-lox661, TetR, CmR	this study
DK-30-cmR-35	DK1622::pSWU30-lox71-cmR-lox662, TetR, CmR	this study
DK-30-cmR-ddW5	DK1622::pSWU30-loxdP-cmR-lox661, TetR, CmR	this study
DK-30-cmR-dd41	DK1622::pSWU30-lox723-cmR-lox661, TetR, CmR	this study
DK-30-cmR-dd35	DK1622::pSWU30-lox d71-cmR-lox661, TetR, CmR	this study
DK-30-cmR-W5-CCK	DK-30-cmR-W5::pBJ113Cre, KanR, TetR, CmR	this study
DK-30-cmR-41-CCK	DK-30-cmR-41::pBJ113Cre, KanR, TetR, CmR	this study
DK-30-cmR-35-CCK	DK-30-cmR-35::pBJ113Cre, KanR, TetR, CmR	this study
DK-30-W5-CCK	DK-30-W5::pBJ113-cre, KanR, TetR	this study
DK-30-41-CCK	DK-30-41::pBJ113-cre, KanR, TetR	this study
DK-30-35-CCK	DK-30-35::pBJ11-cre, KanR, TetR	this study
DK-30-cmR-W5-Pcre	DK-30-cmR-W5,pZJY41-cre, KanR, TetR, CmR	this study
DK-30-cmR-41-Pcre	DK-30-cmR-41,pZJY41Cre, KanR, TetR, CmR	this study
DK-30-cmR-35-Pcre	DK-30-cmR-35,pZJY41Cre, KanR, TetR, CmR	this study
DK-W5	DK-30-W5, TetR	this study
DK-41	DK-30-41, TetR	this study
DK-35-2	DK-30-35-2, TetR	this study
DK-p12lox712	DK1622::pBJ113-12lox712, KanR	this study
DK-12lox712	DK1622::lox712	this study
DK-12lox712-p14lox661	DK1622::lox712::pBJ113-14lox661, KanR	this study
DK-12lox712-14lox661	DK1622::lox712::lox661	this study

**Table 3:** Plasmids used in this study.

Name	Description	Reference or Source
pUC19	pBR322 ori, AmpR	[29]
pNG10A	P*_cuoA_*TetR from pUC19	[22]
pMAT3	P*_cuoA_* Mx8 attPintegraseTetR from pSWU30	[22]
pBJ113	pBR322 ori,*gal*K; KanR	[30]
pSWU30	pBR322 ori, Site-specific integration vector with Mx8 attBintegration site, TetR	[31]
pZJY41	pMF1 ori, pBR322 ori, KanR	[26]
pUC19-cre	pBR322 ori, native *cre* geneAmpR	this study
pUC57-cre(a)	pBR322 ori, codon-optimized artificial cregene, AmpR	this study
pUC19-P*_cuoA_*-*cre*	native *cre* gene promoted by P*_cuoA_*, AmpR	this study
pBJ113-P*_cuoA_*-*cre*		this study
pPCA2	pBJ113-P*_cuoA_*-cre-lox-Frag-lox	this study
pSWU30-Cm^R^-W5	pSWU30, contained the cassette of loxP-cmR-loxP, TetR, CmR	this study
pSWU30-cmR-41	pSWU30, contained the cassette of lox712-cmR-lox661, TetR, CmR	this study
pSWU30-cmR-35	pSWU30, contained the cassette of lox71-CmR-lox662, TetR, CmR	this study
pSWU30-cmR-ddW5	pSWU30, contained the cassette of loxdp-cmR-lox66, TetR, CmR	this study
pSWU30-cmR-dd41	pSWU30, contained the cassette of lox723-cmR-lox661, TetR, CmR	this study
pSWU30-cmR-dd35	pSWU30, contained the cassette of lox723-cmR-lox662, TetR, CmR	this study
pMF1-cre-kanR	pMF1ori, pBR322 ori, native *cre* gene driven by P*_cuoA_*, KanR	this study
pMF1-cre-tetR	pMF1 ori, pBR322 ori, native *cre* gene driven by P*_cuoA_*, TetR	this study
pMF1-cre-cmR	pMF1 ori, pBR322 ori, native *cre* gene driven by P*_cuoA_*, CmR	this study
pMF1-cre(a)-kanR	pMF1ori, pBR322 ori, artificial *cre* gene driven by P*_cuoA_*, KanR	this study
pMF1-cre(a)-tetR	pMF1 ori, pBR322 ori, artificial *cre* gene driven by P*_cuoA_*, TetR	this study
pMF1-cre(a)-cmR	pMF1 ori, pBR322 ori, artificial *cre* gene driven by P*_cuoA_*, CmR	this study
pBJ113-12	Two fragments amplified by two primer pairs contained one lox712 (S12uu-130F, S12uu-1638R2; S12uF2, S12uR2) were cloned into EcoRI, XbaI, HindIII sites of pBJ113, respectively	this study
pBJ113-14	Two fragments amplified by two primer pairs contained one lox661 (S14E-451F, S14E-1457R; S14E-2057F, S14E-3649R) were recombined with linearized pBJ113 by Red/ET	this study

## Supplementary Materials

The following are available online at http://www.mdpi.com/2218-273X/8/4/137/s1, Table S1: Oligonucleotides used in this study. The sequence of codon-optimized artificial *cre* gene cloned in pUC57.Figure S1: Construction of self-replicative plasmids for expression of native *cre* gene or artificial *cre* gene.
