# Supplementary material for: Genome Editing in Model Strain Myxococcus xanthus DK1622 by a Site-Specific Cre/loxP Recombination System
Source: Biomolecules. 2018 Nov 6;8(4):137. doi: 10.3390/biom8040137 (PMC6316027; doi:10.3390/biom8040137)
Supplement: Supplementary file 1 [file biomolecules-08-00137-s001.pdf]

Table S1 primers used in this study.

oligo name	sequence (5’-3’) †
For PCR & cloning	
PcuF	cgGAATTCTTCAACGGCATTCATGGGGCTTCC
PcuR	ggGGTACCCATGAAGCCTCTTCACGAATGGATG
CreF2	ggGGTACCATGTCCAATTTACTGACCGTAC
CreBT1R	gcTCTAGAgcATAAAACGAAAGGCCCAGTCTTTCGACTGAGCCTTTCGTTTTATaaCTAATCGCCATCTTCCAG
Cuo-Lox661-496F	gcTCTAGAgcATAACTTCGTATAGGGTAGGCTATACGAAcggtaCTGTTCGGCGTGCTCATCGTGG
Cuo-Lox712-1578R	GCTGCAGCtaccgTTCGTATAGCCTACCCTATACGAAGTTATTCATTCCTGGGCGGCGTTCC
CmR-LoxPFClaI	ccATCGATggatccATAACTTCGTATAGGGTAGGCTATACGAAcggtaTTCACGCTGCCGCAAGCACTC
CmR-LoxPREcoRV	cgGATATCcgctgcagtaccgTTCGTATAGCCTACCCTATACGAAGTTATACTTATTCAGGCGTAGCACCAGGC
CmR-Lox71F BamHI	cgggatcctaccgTTCGTATA*GCATACTA*TATACGAAGTTAT TTCACGCTGCCGCAAGCACTC
CmR-Lox662R XbaI	gctctagataccgTTCGTATA*GCATACAT*TATACGAAGTTAT ACTTATTCAGGCGTAGCACCAGGC
CmR-WloxPBamHI	cgggatccATAACTTCGTATAATGTATGCTATACGAAGTTAT TTCACGCTGCCGCAAGCACTC
CmR-WloxPXbaI	gctctagaATAACTTCGTATAGCATACATTATACGAAGTTAT ACTTATTCAGGCGTAGCACCAGGC
DDW5F2	ACATTATACGAACGGTAACTTATTCAGGCGTAGCACCAGGC
DDW5R	ATGCTATACGAACGGTATCTAGAGTCGACCTGCAGGC
DD41F	AGGCTATACGAACGGTATTCACGCTGCCGCAAGCACTC
DD41R	ACCCTATACGAACGGTAGGATCCTCTAGAGTCGACCTGC
DD35F2	ATGCTATACGAACGGTAACTTATTCAGGCGTAGCACCAGGC
DD35R	ACTATATACGAACGGTATCTAGAGTCGACCTGCAGGC
V41-3979F	CTGTGATGCGTGCAGTCAGAAGAG
V41-4798R	AAGCGGCAGGGTCGGAACAGGA
TetR 1172F	cccccgggGAGTGGTGAATCCGTTAGCGAGGTG
Tet2644R	cccccgggGCAACTTTATCCGCCTCCATCC
CmRF	CCCCCGGGTTCACGCTGCCGCAAGCACTC
CmRR	CCCCCGGGACTTATTCAGGCGTAGCACCAGGC
S12uu-130F	CGGAATTCGCGTTGATGTCGTCCTTCTCCCA
S12uu-1638R2	GCTCTAGACCGCTCCAACTTCTCGTTCCTTCG
S12uF2	GCTCTAGAtaccgTTCGTATAgggTAggCTATACGAAGTTATACCCGAGGAAGGAGTAGGCG
S12uR2	CCAAGCTTAGTCCAAGCGACAGCATCAGC
S12-1237F	TGGGTCATCCGGCTCTGGGACA

S12-2117R	CACGCTCGGCGGTCATCTGCTT
S12-697LPF1	AACCGCAGTCCTTCGTATGTCAGCAC
S12-1263LPF2	GGCGCACAGGTTCGAGTACCCCGTCC
S14-837F	GGGACGACGCAACTGCTGCTCTTC
S14-2188R	TTGCCTCGCACACCTGACCCACTC
For Red/ET Recombination	
S14E-451F	CGCCAGGGTTTTCCCAGTCACGACGTTGTAAAACGACGGCCAGTGAATTCGGCTTCAAACACCTCTTCAACCTGG
S14E-1457R	GCTCTAGAGGCTGGGTGCCATAGACATACGC
S14E-2057F	GCGTATGTCTATGGCACCCAGCCTCTAGAGCATAACTTCGTATAGGGTAGGCTATACGAAcggtaACCGACCTGCTGAAATCAAACCTG
S14E-3649R	GATAACAATTTCACACAGGAAACAGCTATGACCATGATTACGCCAAGCTTGTAGGAGGGTAAGGTGGAACGGAACT
CuMcrF	TCGTGAAGAGGCTTCATGGGTACCATGTCCAACCTGCTGACGG
McrCuR	TCAGCAGGTTGGACATGGTACCCATGAAGCCTCTTCACGAATGG
41McrR	ATTGTTGCGGTCGACTCTAGAGATATCATAAAACGAAAGGCTCAGTCG
Mcr41F	TTTCGTTTTATGATATCTCTAGAGTCGACCGCAACAATCCACGGGCCGTGTCC

>codon-optimized artificial cre gene (abbreviate cre(a) gene) cloned in pUC57
**catATGTCCAACCTGCTGACGGTGCACCAGAACCTGCCGGCGCTGCCGGTCGACG
CGACGTCCGACGAGGTGCGCAAGAA
CCTGATGGACATGTTCCGGGACCGCCAGGCGTTCAGCGAGCACACGTGGAAGAT
GCTGCTGTCCGTGTGCCGCTCGTGGG
CGGCGTGGTGCAAGCTGAACAACCGCAAGTGGTTCCCCGCGGAGCCGGAGGAT
GTGCGCGACTACCTCCTCTACCTCCAG
GCGCGCGGGCTGGCCGTGAAGACGATCCAGCAGCACCTCGGCCAGCTCAACATG
CTCCACCGGCGGTCCGGGCTGCCCCG
CCCCTCCGATAGCAACGCGGTGTCGCTCGTGATGCGGCGGATCCGGAAGGAGAA
CGTGGACGCCGGTGAACGCGCGAAGC
AAGCCCTCGCGTTCGAGCGGACCGATTTCGACCAGGTGCGCTCGCTCATGGAGA
ACAGCGACCGCTGCCAGGACATCCGG
AACCTCGCCTTCCTGGGTATCGCGTACAACACCCTGCTGCGCATCGCCGAGATCG
CCCGGATTCGCGTGAAGGACATCTC
GCGCACGGACGGCGGCCGGATGCTGATCCACATCGGGCGGACCAAGACGCTGG
TGAGCACCGCCGGGGTGGAGAAGGCGC
TCAGCCTCGGGGTGACGAAGCTGGTCGAGCGCTGGATCTCCGTCAGCGGCGTCG
CGGATGACCCGAACAACTACCTGTTC
TGTCGGGTCCGGAAGAACGGCGTGGCCGCGCCCAGCGCCACCAGCCAGCTCTC
GACGCGCGCCCTGGAAGGTATCTTCGA
GGCGACCCACCGCCTGATCTACGGCGCGAAGGACGACTCCGGCCAGCGGTATCT
GGCCTGGTCCGGCCATTCCGCCCGCG**

TCGGCGCCGCGCGCGACATGGCCCGCGCCGGCGTGTCGATTCCGGAAATTATGC
AGGCGGGCGGCTGGACCAATGTCAAT
ATCGTCATGAATTACATCCGCAACCTGGACTCGGAGACGGGCGCGATGGTGCGC
CTGCTGGAGGACGGCGACTGAaaATA
AAACGAAAGGCCCAGTCTTTCGACTGAGCCTTTCGTTTTATgatatcgaattc

1-6 NdeI
4-1035 codon-optimized cre
1036-1081 rrnT1
1082-1087 EcoRV
1088-1093 EcoRI

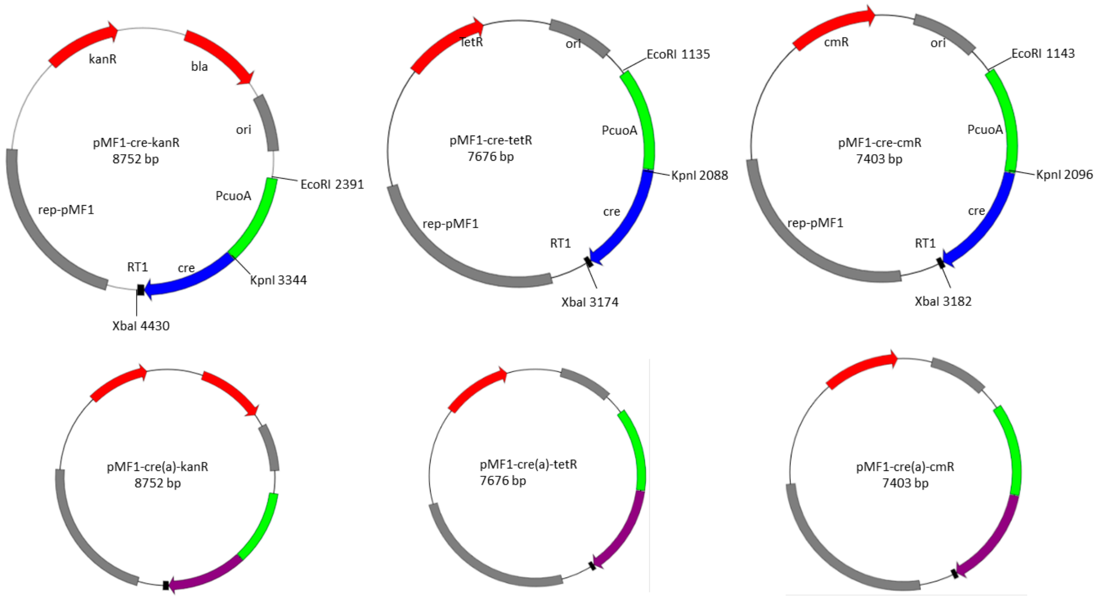

Figure S1. Construction of self-replicative plasmids for expression of native *cre* gene or artificial *cre* gene. Except the pMF1-cre-kanR, five other self-replicative plasmids were constructed. The kanamycin gene and ampicillin gene were replaced by tetR and cmR to obtain plasmid pMF1-cre-tetR and pMF1-cre-cmR, respectively. The native cre was replaced by the artificially synthetic cre gene to obtain the pMF1-cre(a)-kanR, pMF1-cre(a)-tetR and pMF1-cre(a)-cmR.
